# Enhanced Tribological and Bacterial Resistance of Carbon Nanotube with Ceria- and Silver-Incorporated Hydroxyapatite Biocoating

**DOI:** 10.3390/nano8060363

**Published:** 2018-05-24

**Authors:** Aditi Pandey, Anup Kumar Patel, Ariharan S., Vikram Kumar, Rajeev Kumar Sharma, Satish Kanhed, Vinod Kumar Nigam, Anup Keshri, Arvind Agarwal, Kantesh Balani

**Affiliations:** 1 Biomaterials Processing and Characterization Laboratory, Department of Materials Science and Engineering, Indian Institute of Technology Kanpur, Kanpur-208016, Uttar Pradesh, India; aditip@iitk.ac.in (A.P.); anupp7454@gmail.com (A.K.P.); ariharan@iitk.ac.in (A.S.); rksk@iitk.ac.in (R.K.S.); ksatish@iitk.ac.in (S.K.); 2 Department of Mechanical Engineering, Indian Institute of Technology Kanpur, Kanpur-208016, Uttar Pradesh, India; vikramk@iitk.ac.in; 3 Department of Bio-Engineering, Birla Institute of Technology, Mesra, Ranchi-835 215, Jharkhand, India; vknigam@bitmesra.ac.in; 4 Department of Materials Science and Engineering, Indian Institute of Technology Patna, Patna-801103, Bihar, India; anup@iitp.ac.in; 5 Department of Mechanical Engineering, Florida International University, Miami, FL 33172, USA; agarwala@fiu.edu

**Keywords:** hydroxyapatite, ceria, silver, plasma-spraying, antibacterial, cytocompatible, filopodial-protrusions

## Abstract

Pertaining to real-life applications (by scaling up) of hydroxyapatite (HA)-based materials, herein is a study illustrating the role of carbon nanotube (CNT) reinforcement with ceria (CeO_2_) and silver (Ag) in HA on titanium alloy (TiAl6V4) substrate, utilizing the plasma-spraying processing technique, is presented. When compared with pure HA coating enhanced hardness (from 2.5 to 5.8 GPa), elastic modulus (from 110 to 171 GPa), and fracture toughness (from 0.7 to 2.2 MPa·m^1/2^) elicited a reduced wear rate from 55.3 × 10^−5^ mm^3^·N^−1^·m^−1^ to 2.1 × 10^−5^ mm^3^·N^−1^·m^−1^ in HA-CNT-CeO_2_-Ag. Besides, an order of magnitude lower Archard’s wear constant and a 41% decreased shear stress by for HA-CNT-CeO_2_-Ag coating depicted the effect of higher hardness and modulus of a material to control its wear phenomenon. Antibacterial property of 46% (bactericidal) is ascribed to Ag in addition to CNT-CeO_2_ in HA. Nonetheless, the composite coating also portrayed exaggerated L929 fibroblast cell growth (4.8 times more than HA), which was visualized as flat and elongated cells with multiple filopodial protrusions. Hence, synthesis of a material with enhanced mechanical integrity resulting in tribological resistance and cytocompatible efficacy was achieved, thereupon making HA-CNT-CeO_2_-Ag a scalable potent material for real-life load-bearing implantable bio-coating.

## 1. Introduction

Hydroxyapatite (HA) has found its applications in biomedical applications (dentistry and orthopedics) due to its imperative property of biocompatibility and osteoconductivity (and is therefore bioactive) [1,2,3]. Biocompatibility and bioactivity is highest in hydroxyapatite. It is known that hydroxyapatite chemically bonds (directly) with hard tissues [4]. Clinically, HA coatings on bioinert metallic implants [5] have been applied. Despite the good biocompatibility of HA, its poor fracture toughness (<1 MPa·m^1/2^) has restricted its use in orthopedic-load-bearing applications [6]. Therefore, HA coating on metals (higher mechanical strength, so stronger substrate) may provide increased strength and fatigue resistance. A thin coating of hydroxyapatite on the implant surface has been reported to improve on aspects encompassing bone implant contact, fixation of the implant, load-bearing capacity, and the osseointegration process, bone regeneration, and healing [7,8,9]. In this regard, HA is found to achieve a comparable biocompatibility and bioactivity for dental and skeletal implants comprised of titanium and its alloys [5,10]. Titanium and its alloys are commonly used as substrate for implants (orthopedic or dental) because of their lightness, excellent corrosion resistance, and mechanical properties [5], which make them better than other metals. They are biocompatible, but the ions released in the blood stream may have detrimental effects on the patient, leading to inflammation or allergies and toxic reactions [11,12,13]. By coating the titanium surface with a material mimicking the organic and inorganic phase of bone, a physiological transition may be created between its (titanium) non physiological surface and the bone tissue in the vicinity [7,8].

Plasma spraying is a commercially existing technique used for the economic coating of HA for applications in real life [10]. Although new processing and coating techniques are available, their application is limited due to generation of non-biocompatible phases [14], non-homogeneity, degradation, amorphous and higher carbonated HA coating [15], weight loss and residue [16], and functionally restricted scalability [17]. However, micro-cracks and high porosity in plasma-sprayed HA coatings (also having peculiar brittleness of HA, low fracture toughness, making it mechanically inadequate) subject the coatings for delamination from the substrate. For mechanical property enhancement, reinforcement of the HA matrix with a second material is essential, for which the use of several materials like carbon nanotube (CNT), yttria stabilized zirconia (YSZ), alumina (Al_2_O_3_), and silver (Ag) has been reported [18,19,20].

An attractive rare-earth compound, ceria (CeO_2_), known for its antioxidant role [21], and Ag nanoparticles (NPs), a bactericidal agent, known to combat post-implant infections [22], have been used in an earlier work [23] synergistically into the HA matrix to achieve antioxidant, antibacterial, and cytocompatible spark plasma sintered composites for orthopedic applications. There was an enhancement in hardness by 1.6 times (from 5 GPa to 9 GPa), elastic modulus by 1.4 times (from 121 GPa to 168 GPa), and fracture toughness by 4.2 times fracture toughness (from 0.2 MPa·m^1/2^ to 0.9 MPa·m^1/2^) by Ag and CeO_2_ reinforcement in HA. While the mechanical property improved (*H*, *E*, and *K_IC_*), it was observed that it reached only to 0.9 MPa·m^1/2^, which is again lesser than that needed (toughness of bone, 2.3 MPa·m^1/2^) [24]. Therefore, addition of CNT into the HA matrix can be a possible route attributed to their exceptional mechanical properties (Young’s modulus) and physical properties as well as good biocompatibility [25,26]. The CNT is also found to possess a hydrophobic nature (may be essential for protein and cell adsorption [23]) and are lightweight [27]. CNT-based composite materials exhibit biocompatibility without cytotoxicity as an important property for biomedical orthopedic and cardiovascular applications [28] due to its reported bioactivity [29,30,31]. Plasma- sprayed HA-4 wt % CNT coatings depicted unrestricted human osteoblast hFOB cell growth and also showed apatite precipitation and mineralization on the CNT surface [25], which acts as crystallization site for apatite crystal growth. Balani et al. also demonstrated that HA-4 wt % CNT plasma sprayed coating was found to have uniformly distributed undamaged CNTs and fracture toughness enhanced by 56% [25]. HA-4 wt % CNT composites and HA-Al_2_O_3_-CNT coatings have also shown a wear resistance enhancement of 66% [32] and 68 times [33], respectively, when compared to that of HA due enhanced hardness, modulus, and fracture toughness. Hence, CNTs incorporation may improve the mechanical properties of HA coatings without affecting their biocompatible properties [34,35,36].

Therefore, the novelty of the present work lies in fabricating a composite coating comprising CNT reinforcement along with CeO_2_ and Ag NPs in HA on a titanium alloy (TiAl6V4) substrate to achieve (i) higher mechanical properties (hardness and toughness) (ii) without compromising the antibacterial and cytocompatible efficacies in comparison to HA, thereby extending from laboratory scale to real-life improvements in the life of body inserts and implants for human healthcare.

## 2. Materials and Methods

### 2.1. Processing

Irregular HA (abbreviated as HA, 10–50 µm), blended with 4 wt % CNT (HA-4C, 40–70 nm diameter, 95%+ purity, 2 µm length, Nanostructured and Amorphous Materials Inc., Houston, TX, USA), 4 wt % CNT-5 wt % CeO_2_ (HA-4C-5Ce, CeO_2_ NPs were of 60–100 nm, from Inframat Advanced Materials, Manchester, CT, USA), and 4 wt % CNT-5 wt % CeO_2_-5 wt % Ag (HA-4C-5Ce-5Ag, Ag NPs were of 80–100 nm, procured from Inframat Advanced Materials, Manchester, CT, USA) in a jar mill for 3 h. Plasma sprayed coatings were deposited on TiAl6V4 substrate using Praxair SG 100 gun.

Following were the parameters used during plasma spraying: power (24 kW), primary gas Ar (30 slm), secondary gas He (28 slm), carrier gas Ar (25.5 slm), standoff distance (100 mm), and feed rate (3 grams per minute). The coating thickness was targeted to be around ~100–120 µm for all the coatings.

### 2.2. Phase, Microstructural Characterization and Mechanical Characterization

The samples were cloth polished by final alumina suspension (particle size, 0.22 μm). X-ray diffraction (XRD, Rich-Seifert, Mannheim, Germany), 2000D diffractometer, at 25 kV and 15 mA, having Cu-K*α* (*λ* = 1.541 Å) radiation at a scan rate of 0.5°/min, and a step size of 0.02°, was used for the phase analysis studies. The cross-section of the samples was examined for the morphological characterization, and the coating thickness was thereby determined via Scanning Electron Microscopy (SEM; SUPRA40VP, Carl Zeiss NTS GmbH, Oberkochen, Germany). Raman spectroscopy (WITec GmbH, Ulm, Germany, Alpha 300) was utilized for plasma-sprayed coatings to investigate the presence of CNTs.

The polished samples were subjected to Vickers microhardness tester with a load of 200 g and a dwell time of 10 s for estimating hardness of the plasma sprayed HA coatings. In-order to calculate the fracture toughness, a load of 500 g was used for 10 s dwell time. For radial crack length measurement, SEM images of the indents with the cracks were taken.

### 2.3. Tribological Analysis of HA-Based Coatings

The friction and wear testing was performed by the ball on disc method by Nanovea Tribometer. For a typical experiment, a 6 mm diameter stainless steel ball was rotated on the samples (in a circular wear track). Rectangular shaped samples of 5 mm × 5 mm × 2 mm are clamped on sample stage. A load of 10 N was applied with sliding speed of 150 rpm and for a duration of 90 min. The worn surface profiles were obtained by 3D optical surface profilometer (Contour GT-K, Bruker, Tucson, AZ, USA) used for the estimation of wear volume by Equation (1) [37]. The extent of damage and worn surfaces of HA-based coatings were further imaged by scanning electron microscope (W-SEM, JSM-6010LA, JEOL, Freising, Germany).

(1) $$Wear\;volume=\pi \;\left(d_{2}-d_{1}\right)\;h\times w$$

where *d*_2_ and *d*_1_ are the outer and inner diameter of wear track, respectively, *h* is the depth, and *w* is the width of wear track.

### 2.4. Wettability and Protein Adsorption

The wettability (hydrophobicity/hydrophilicity) of the surface was measured by water contact angle on the surface of each sample (*n* = 3, with repetition of ten times on each) by a contact angle goniometer (Data-physics Contact Angle System OCA). The protein (bovine serum albumin, BSA) adsorption behavior was quantified by a standard protocol according to the bicinchoninic acid assay kit (Cat. # 786-570,786-571, G-biosciences). Samples (*n* = 3) in a 24-well plate were introduced with protein concentration (of 2 mg/mL) and incubated at 37 °C for 24 h. The non-adhered proteins were washed (after incubation) by rinsing with 1XPBS. ELISA plate reader was used to evaluate the concentration of the adsorbed proteins at absorbance of 540 nm.

### 2.5. Antimicrobial Test

The antibacterial activity of the HA-based coatings was studied by Gram-negative bacteria (*E. coli*; MCC2079). The samples were polished, autoclaved, rinsed with ethanol, and washed thoroughly with 1XPBS before seeding. In a 24-well culture plate, 200 μL bacterial solutions (0.1 optical density) were seeded on each of the samples (triplicate samples tested for three different times). The samples were then incubated at 37 °C for 4 h, after which they were washed thoroughly with 1XPBS to remove the non-adhered bacterial cells. The samples were finally analyzed qualitatively (by scanning electron microscopy) and quantitatively (by MTT assay). The adhered bacterial cells were fixed first with 3% glutaraldehyde (for 20 min), followed by 0.1 M sodium cacodylate (for 15 min), and then 0.1 M sucrose (for 15 min). After rinsing with 1XPBS again, samples were dehydrated using serially diluted alcohol series (for 10 min each) and dried finally by hexamethyldisilazane (HMDS, critical point dryer). Samples were eventually sputter-coated with gold for the SEM imaging (SEM; SUPRA40VP, Carl Zeiss NTS GmbH, Oberkochen, Germany). 

In order to determine metabolic activity/viability of bacterial cells, MTT assay (MTT: (3(4,5-dimethylthiazol-2-yl)-2,5-diphenyl tetrazolium bromide), Amresco, Life Science Research Product & Biochemicals, Logan, UT, USA) was utilized. A 1:10 ratio of MTT: PBS was added onto each cultured sample and incubated at 37 °C for 2 h. MTT forms the formazan crystals upon reacting with viable cells, which are further dissolved by dimethyl sulphoxide (DSMO) producing a purple color. The absorbance of the purple colored solution was read at 570 nm, by using an ELISA plate reader (ultraviolet-vis BioTek, Winooski, VT, USA) [38]. 

### 2.6. Cytocompatibility Test

The L929 mouse fibroblast (L929 NCCS Pune) were used for conducting in vitro tests. The cells were cultured in complete medium consisting of Dulbecco’s modified eagles–base medium supplemented with fetal bovine serum (Cat no. F2442, Sigma Aldrich, Munich, Germany) and 1% of antibiotic solution (penicillin, Cat no. A5955, Sigma Aldrich, Germany). Cultured cells were incubated at 37 °C, 5% CO_2_, and 95% humidity, after which a sub-confluent cells (monolayer) was expanded by trypsin-EDTA solution (Cat no. T4049, Sigma Aldrich, Germany). For a typical experiment, pellets (*n* = 3) were seeded with a cell density of 2 × 10^4^ cells/mL in a 24 well plate and incubated for seven days. Scanning electron microscopy was utilized to image cell morphology on the sample surface. Quantification of cellular metabolic activity was performed by MTT assay at days 1, 3, and 7. The protein adsorption and the MTT assay experiments were repeated three times.

## 3. Results

### 3.1. Phase Analysis

**Figure 1:**
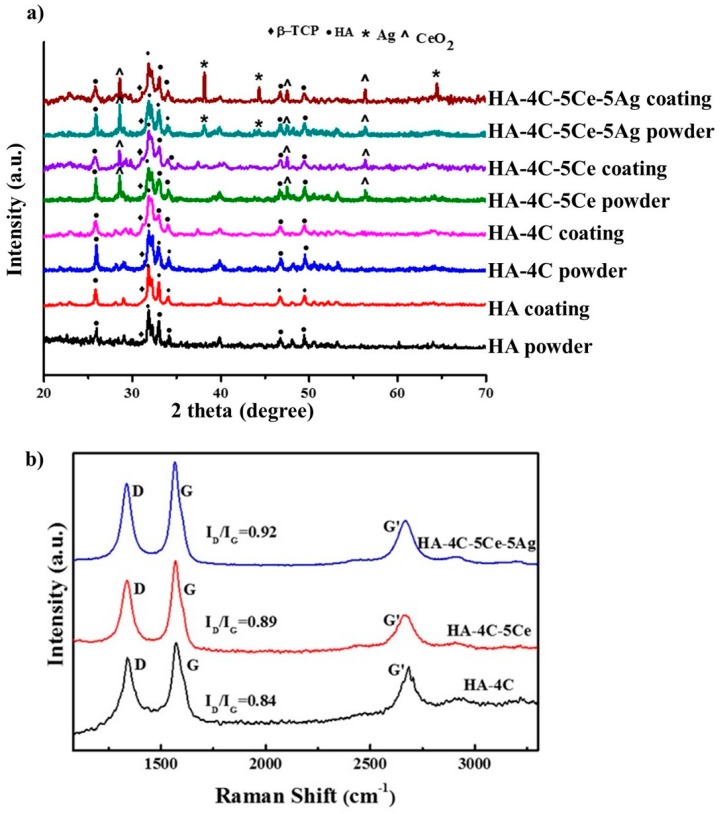
(**a**) X-ray diffraction (XRD) pattern of powders and coatings of hydroxyapatite (HA), HA-4C, HA-4C-5Ce, and HA-4C-5Ce-5Ag on TiAl6V4 substrate and (**b**) Raman spectra of carbon nanotube (CNT) containing composite coatings.

The phase characterization (XRD pattern) of hydroxyapatite, ceria, and silver powder, and their coatings on titanium alloy substrate are presented in Figure 1a. As can be seen from Figure 1, no additional peaks are obtained in XRD pattern except β-TCP at 2θ of 31.02° corresponding to plane (0210), indicating that no major reaction has occurred in the constituent materials of coatings. The peaks corresponding to ceria can be observed from the planes (111), (311), and (220), with 2θ value of 28.61°, 56.48°, and 47.59° and the peaks for silver seen from planes (111), (200), (220) with 2θ value of 38.23°, 44.34°, and 64.51°.

Figure 1b shows the Raman analysis of samples containing CNT. The D band at 1340 cm^−1^ and G band at 1572–1582 cm^−1^ in the spectra that corresponds to the characteristic of CNTs confirm its presence in the coatings, with ID/IG ratio ranging between 0.84–0.92. The D band and G bands correspond to defects in the graphene sheets and stretching mode of graphitic backbone, respectively.

### 3.2. Microstructural Characterization and Mechanical Properties

**Figure 2:**
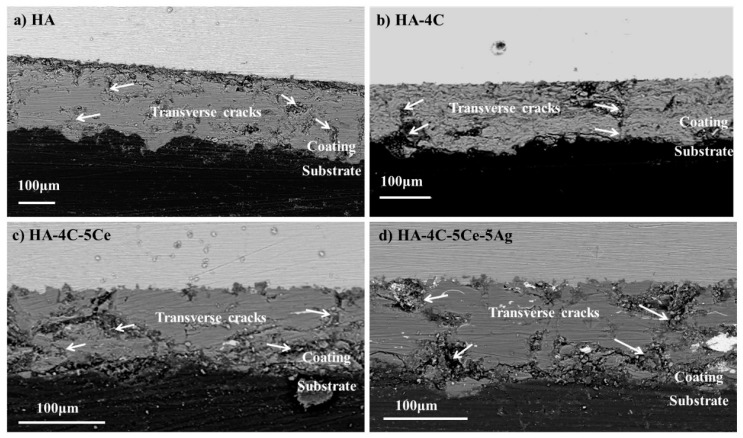
Cross sectional view of plasma sprayed coatings of (**a**) HA, (**b**) HA-4C, (**c**) HA-4C-5Ce, and (**d**) HA-4C-5Ce-5Ag.

The SEM images of cross sections of plasma sprayed coatings of HA, HA-4C, HA-4C-5Ce, and HA-4C-5Ce-5Ag on TiAl6V4 substrate are shown in Figure 2a–d which depicts a lamellar structure showing no delamination from the TiAl6V4 substrate. Coatings are continuous, uniform and adherent. Thickness of the HA coating is observed to be 100–130 µm (Figure 2) due to which have not been found to chip off or delaminate after sample processing. All the coatings possess a similar nature with transverse cracks, with µm size porosity distributions.

The mechanical properties of the coatings are presented in Table 1. The hardness of HA was obtained to be 1.88 GPa, which increased to 3.95 GPa with the addition of CNT (HA-4C) as also reported in the literature [25,32,33]. Increased hardness was recorded in HA-4C-5Ce-5Ag (5.8 GPa) in comparison to HA-4C-5Ce (4.13 GPa) due to Ag addition in the composite. Furthermore, the elastic modulus of the composite coatings was estimated by the rule of mixture, wherein, 110 GPa for pure HA, 165.3 GPa for HA-4C, 170.9 GPa for HA-4C-5Ce, and 171.4 GPa for HA-4C-5Ce-5Ag, again following the similar trend reported in the literature [22,23].

**Figure 3:**
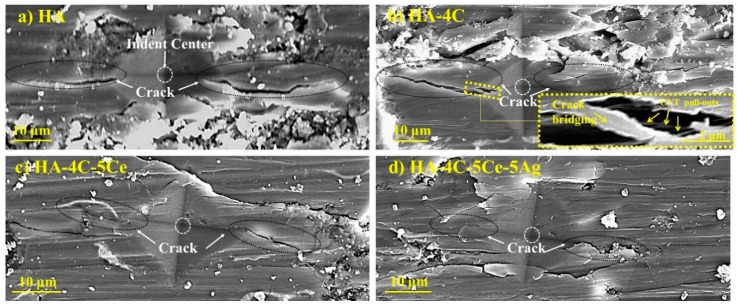
Propagating radial crack from the indent in plasma sprayed coatings of (**a**) HA, (**b**) HA-4C (inset depicting the crack showing CNT pull-outs and crack bridging phenomenon), (**c**) HA-4C-5Ce, and (**d**) HA-4C-5Ce-5Ag.

The fracture toughness was calculated by the Anstis’ Equation (2) [39].

(2) $$K_{IC}=0.016\;{\left(E/H\right)}^{1/2}\times F/C^{3/2}$$

where *H* and *E* are the hardness and elastic modulus of the coatings, *F* is the load at which the cracks were generated, and *C* is the critical crack length for all HA-based samples (shown in Figure 3a–d) from the indent center. The average fracture toughness of pure HA coating was found to be 0.68 MPa·m^1/2^ and 0.89 MPa·m^1/2^ for HA-4C, 1.12 MPa·m^1/2^ for HA-4C-5Ce, and 2.12 MPa·m^1/2^ for HA-4C-5Ce-5Ag. It can be observed that the fracture toughness increases by ~1.3 times with CNT addition, ~1.6 times with CNT and CeO_2_ addition, and ~3.1 times with synergistically reinforced CNT-CeO_2_-Ag. The toughening mechanisms in CNT is attributed to phenomena of CNT assisting in crack-bridging, crack-deflection, and CNT pull-outs [22], as observed from inset of Figure 3.

**Figure 4:**
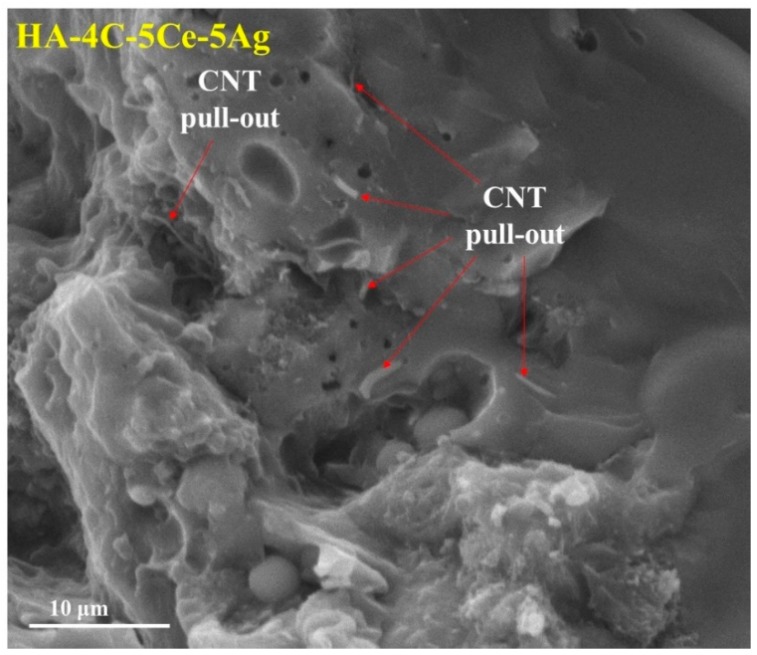
Fractured surface of HA-4C-5Ce-5Ag showing the CNT pull-outs.

It can be noticed that the fracture toughness in CNT-CeO_2_-incorporated samples does not deteriorate (marginal enhancement, despite the brittle nature of CeO_2_ ceramic) in comparison to HA-CNT. In the case of HA-4C-5Ce-5Ag, the Ag NPs melt/liquefy due to its lower melting point of 960 °C, and therefore it is realized that they contribute to the volumetric toughening by their homogenous distribution [22,40] in the HA matrix. This explains the increased toughness by Ag incorporation into the HA-4CNT coating. Furthermore, propagating crack tip (from stress) is shielded by the generated compressive stresses upon alloying yttria-doped tetragonal zirconia ceramics with CeO_2_ yielding higher toughness [41,42], and deformed Ag NPs (added into HA-4CNT matrix) absorb enhanced energy in a propagating crack (by crack tip blunting and bridging) [22]. The fractured surface of HA-4C-5Ce-5Ag (Figure 4) shows the presence of CNT, which is retained in the sample after processing. It can be inferred that isolated interfacial delamination of CNT or toughening provided by CeO_2_ NPs contributing to enhanced toughness is feeble when compared to the highest toughness obtained for HA-4C-5Ce-5Ag layer, which is elicited by synergistic effect of the delaminated CNTs and the volumetric toughening rendered by the effect of Ag.

### 3.3. Tribological Studies of HA-Based Coatings

**Figure 5:**
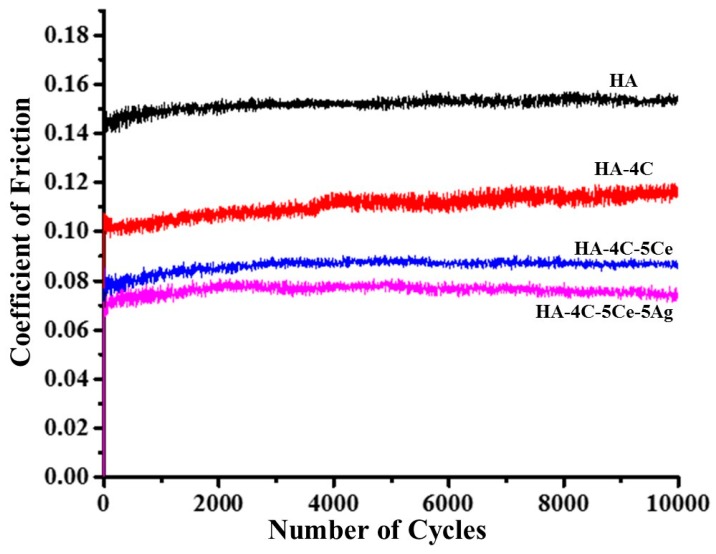
Plot representing coefficient of friction (COF) as a function of number of cycles during ball-on-disc of HA, HA-4C, HA-4C-5Ce, and HA-4C-5Ce-5Ag composites.

The coefficient of friction (COF) obtained during the tribology of HA-based coatings was found to decrease with CNT CeO_2_ and/or Ag reinforcement (Figure 5). The COF during the steady state was found to be 1.5, 1.8, and 2 times lower than that of HA (COF: 0.16) for HA-4C (COF: 0.11), HA-4C-5Ce (COF: 0.09), and HA-4C-5Ce-5Ag (COF: 0.08), respectively. The reduced COF for CNT-incorporated samples can be attributed to the lubrication provided by a peeled-off graphene layer from CNT (by the applied shear force) and its deposition on the wear track [43]. Furthermore, CeO_2_ and Ag NPs reinforcement into the HA matrix have also been reported to cause a 4.4 times reduction in COF (due to enhanced 1.6 times hardness and 4.2 times toughness) when compared to that of HA [44]. Factoring in the combined effects of appreciated mechanical properties (*E*, *H*, and *K_IC_*) by the reinforcing phases may be an important aspect decreasing the coefficient of friction.

Hertzian contact diameter (HCD) for counter body (stainless steel ball) and substrate (wear behavior of material) configuration is estimated by the following Equation (3) [45]:

(3) $$D={\left(\frac{3RF_{n}}{4E^{*}}\right)}^{\frac{1}{3}},\;\mathrm{where}\;\frac{1}{E^{*}}=\frac{1-\nu_{1}^{2}}{E_{1}}+\frac{1-\nu_{2}^{2}}{E_{2}}$$

where *F_n_* is the load, *R* is the radius of the counter-body (ball), and *ν*_1_ and *ν*_2_ are the Poisson’s ratio and *E*_1_ and *E*_2_ correspond to the Young’s modulus of the steel ball and the substrate, respectively.

The HCD values for HA was estimated to be ~66 μm, which decreased to ~61 μm for all the other composites (HA-4C, HA-4C-5Ce, and HA-4C-5Ce-5Ag), owing to higher elastic modulus (Table 1) (as HCD is primarily a function of modulus of mating material). Therefore, it can be said that the higher HCD (more sliding surface area, and thus more counter body–sample interaction) resulted in more surface damage/wear [45]. The change in HCD was not found to be very significant but is not the only criteria in determining wear resistance. Further, to estimate the generated stress levels relating to the damage caused, the Hertzian contact pressure (*HCP*) and the shear stress was calculated by the following Equation (4) [44,45]:

(4) $$HCP={\left(\frac{6F_{n}{\left(E^{*}\right)}^{2}}{3.14^{3}\times R^{2}}\right)}^{\frac{1}{3}}$$

where *F_n_* is the applied load and *R* is the counter body radius. The value of E* is reported in Table 2 (from *HCD* calculations of fretting), and the shear stress (*τ*) can be computed from the following relation:

(5) τ=HCP×COF

The shear stress for HA coating was estimated to be 173.7 MPa, which reduced to 140.6 MPa (19% reduction) with CNT incorporation, and 115 MPa (33% reduction) and 102.9 MPa (41% reduction) with CNT-CeO_2_ and CNT-CeO_2_-Ag reinforcement, respectively. The attained wear resistance is also higher (25.9 times) for the CNT, CeO_2_, and Ag-incorporated coatings attributed to the contact stresses leading to effective densification of the graphitic layers from CNT, providing lubrication and thus a decreased wear rate (Table 2). The high values of the pressure created during the wear of HA (no lubrication provided due to absence of CNTs) has resulted highest damage and thus eliciting a maximum wear rate (176.1 × 10^−5^ mm^3^·N^−1^·m^−1^) amongst the coatings.

**Figure 6:**
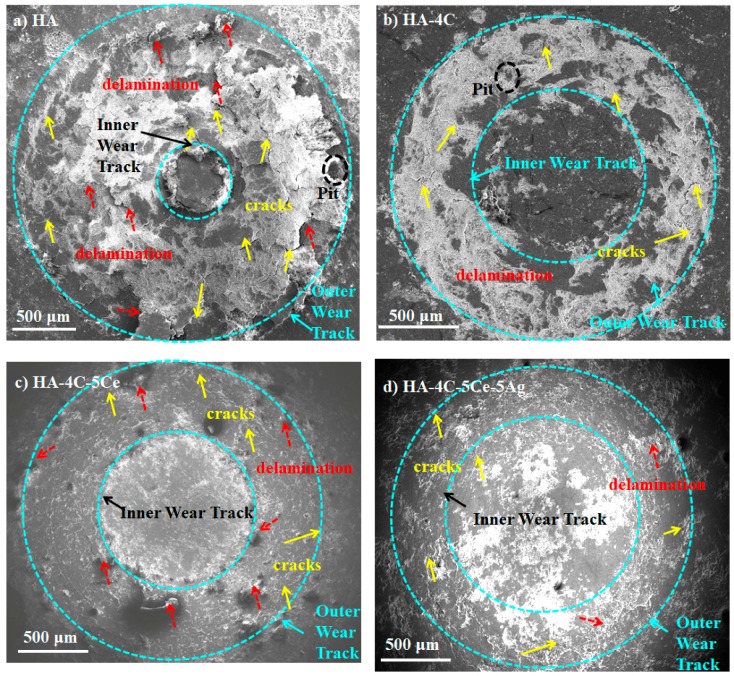
Micrographs showing the worn surface scar of (**a**) HA, (**b**) HA-4C, (**c**) HA-4C-5Ce, and (**d**) HA-4C-5Ce-5Ag (yellow and red arrows show cracks and delamination, respectively).

The scanning electron micrographs of the worn surfaces after the wear test of HA-based composite coatings are represented in Figure 6a–d. Severe damage (a worn surface) can be seen by the extensive delamination, cracks, and pits in the HA coatings (Figure 6a). However, it was realized that with CNT, CeO_2_ and/or Ag reinforcement, restricted damage occurred due to recognized smoothening (Figure 6b–d, roughness after wear in Table 2), which may be due to the lubrication from the graphene layers of CNT and the abrasion resistance imparted by the coatings [46]. The synergistically reinforced HA-4C-5Ce-5Ag (Figure 6d), exhibited least damage (highest surface smoothening effects). The high shear stress–initiated (140.6 MPa) CNT pull-outs in HA-4C (also due to lower *H* and *K_IC_* than HA-4C-5Ce and HA-4C-5Ce-5Ag) agglomerated and discontinuous graphene tribofilm, as also reported earlier [43]. This tends to cause a disparity of surface smoothening in comparison to HA-4C-5Ce (roughness, 6 µm) and HA-4C-5Ce-5Ag (lesser CNT pull-outs, resulting in lowered graphene layers agglomeration and thereby the smoothening is higher, with a roughness of 5 µm) [43,46]. Therefore, the wear resistance attained in case of HA-4C is lower (discontinuous tribofilm) than HA-4C-5Ce and HA-4C-5Ce-5Ag (smoother tribofilm, roughness of 5 µm). However, the wear resistance in HA-4C is higher than HA due to the tribofilm deposition (though uneven, rough, 10 µm), which (tribofilm) is not there in pure HA coating. Thus, it is the combined effect of shear stress (leading to matrix smoothening of CNTs by its graphene layers) and mechanical integrity (*H* and *K_IC_*, Table 1) that results in the maximum wear resistance in HA-4C-5Ce-5Ag coating. The 25.9 times higher wear resistance (Table 2) is further supported by the decreased lateral force generated at same normal force, which is attributed to the reduced COF and reduction of damage (worn surface area) in the wear track [32].

**Figure 7:**
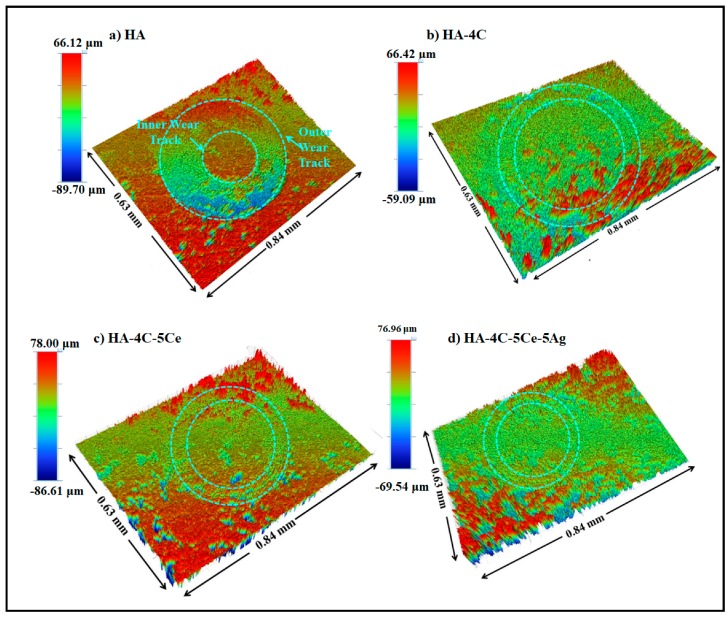
Three-dimensional images (**a**–**d**) observed by surface profilometery of the worn surface scar of HA, HA-4C, HA-4C-5Ce, and HA-4C-5Ce-5Ag.

The wear track profiles and the occurred damage (i.e., wear volume) were determined by optical profilometry (Figure 7a–d). Table 2 presents the reduction in the worn volume with CNT, CeO_2_, and/or Ag incorporation in HA. The wear volume of 0.062 ± 0.007 mm^3^ was obtained in HA (Figure 7a), which decreased to 0.023 ± 0.005 mm^3^ in HA-4C (by 3 times, Figure 7b). Further a remarkable reduction of 20 times was found to be in HA-4C-5Ce sample (wear volume, 0.003 ± 0.001 mm^3^, Figure 7c) with the least volume of 0.002 ± 0.001 mm^3^ obtained in HA-4C-5Ce-5Ag coating, Figure 7d, which is 30 times lesser than that of HA. 

Wear rate (*W_R_*) was calculated using the following Equation (5) and shown in Table 2.

(6) $$W_{R}=\frac{W_{v}}{Load\times S}$$

where *S* is the sliding distance. The wear rate was estimated to be highest for HA, 176.1 × 10^−6^ mm^3^·N^−1^·m^−1^, which was reduced by 3 times to 54.7 × 10^−6^ mm^3^·N^−1^·m^−1^ for HA-4C, with a further reduction of 18 times to 9.7 × 10^−6^ mm^3^·N^−1^·m^−1^ for HA-4C-5Ce. A reduction of 26 times in the wear rate for HA-4C-5Ce-5Ag coating is attributed to an increased hardness and toughness, as shown in Table 1. The achieved wear rate in the coatings can also be correlated with the tribofilm formation (and thickness) with CNT, CeO_2_, and Ag reinforcement as seen by the SEM images in Figure 5b–d.

The relation of the bulk hardness (*H*) of the material with the wear volume can be studied using the Archard equation (to evaluate the wear constant, *k*) via Equation (6).

(7) $$k=\frac{W_{v}\times H}{P\times L}$$

where *H* is the hardness of coatings, *P* is load applied, and *L* is sliding distance. Upon application of load, the resistance in wear loss by the hardness of the material portrays the wear constant. The wear constant of 13.8 × 10^−3^ was estimated for pure HA coating, illustrating highest wear loss. For HA-4C, the wear constant was found to be decreased to 6.7 × 10^−3^. The extremely low values of wear constant, of 1.3 × 10^−3^ and 1.2 × 10^−5^, was computed for HA-4C-5Ce and HA-4C-5Ce-5Ag, respectively, owing to enhanced hardness (due to effect of synergistic addition of CNT, CeO_2_ and Ag), which thereupon resulted in the least wear rate (wear resistance increased by 18 and 26 times by HA-4C-5Ce and HA-4C-5Ce-5Ag, respectively).

**Figure 8:**
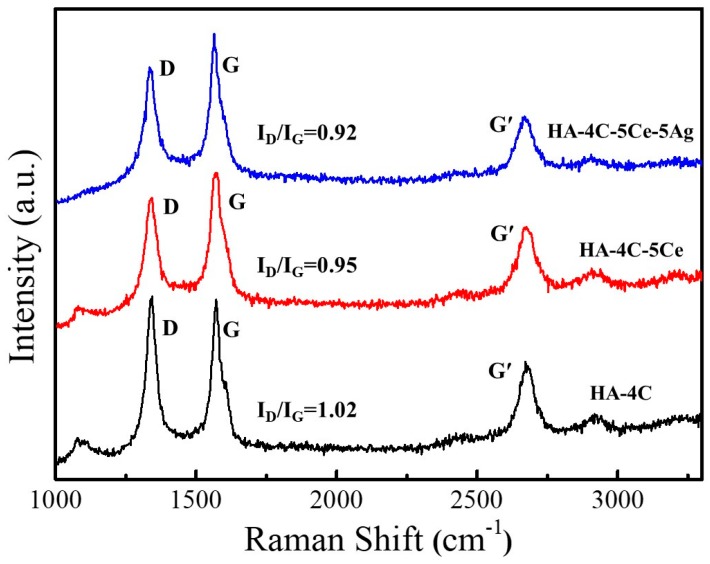
Raman spectra after wear of HA-4C, HA-4C-5Ce, and HA-4C-5Ce-5Ag plasma-sprayed coatings.

In order to understand the effect of wear on the CNT reinforced plasma sprayed coatings, Raman spectroscopy was utilized. The I_D_/I_G_ ratio in the coatings after wear (Figure 8) was increased from 0.95 to 1.02 in comparison to that before wear ranging from 0.84 to 0.92 (Figure 1b), which may be attributed to an increased defect in the carbon structure by the shear stress generated during the wear phenomenon (causing CNT distortion and peeling of its graphene layers). It can be observed from the Raman spectra that a maximum of 1.01 I_D_/I_G_ is obtained in the HA-4CNT coating, which may be attributed to highest 140.6 MPa shear stress upon wear due to lowest *H* and *K_IC_* among all CNT reinforced coatings). However, a significant wear resistance (3 times) is obtained in comparison to pure HA due to the peeled off graphene layers (leading to its agglomeration).

### 3.4. Wettability Studies and Its Role in Protein Adsorption of HA-Based Coatings

The water contact angles (WCA) on HA-based coatings is shown in Table 3. A contact angle of 74° was obtained on HA (hydrophilic surface), which increased to 104° on HA-4C and 112° on HA-4C-Ce coating, showing an increase in hydrophobicity by CeO_2_ and/or CNT reinforcement. This is due to the inherent hydrophobic nature of CNT [47] and CeO_2_ [48] (shielding effect of the 4f orbital by outer 5s^2^p^6^ shell [48,49,50]). When considering HA-4C-Ce-Ag substrate, the contact angle was observed to be 97° because of comparatively lesser hydrophobicity of silver [23,51] in comparison to CNT or CeO_2_. However, the surface was found to be hydrophobic when compared to the hydrophilic HA.

It is known that surface properties like hydrophobicity and electronic charge affect protein adsorption and cell adhesion up to 48 h [52,53]. From the protein adsorption presented in Table 3, it can be observed that BSA adsorption of 2.1 μg·mm^−2^ was obtained on pure HA coating. With an increase in hydrophobicity, the protein adsorption was found to increase to 3.4 μg·mm^−2^ (1.6 times) for HA-4C. A BSA adsorption of 2.0 times than HA (4.2 μg·mm^−2^) was obtained on HA-4C-5Ce-5Ag (despite its lower contact angle than HA-4C) due to the combined action of hydrophobicity (of CNT-CeO_2_-Ag) and chemistry of Ag (spontaneous adsorption of proteins on surface of a silver-coated implant [54,55], and specific binding of silver with -SH groups of BSA protein [56,57]). It can be noticed that HA-4C-5Ce elicited a maximum adsorption of 5.0 μg·mm^−2^ (2.4 times higher than that on pure HA) is again attributed to the effect of its highest hydrophobicity. The role of hydrophobicity (solely acting as the main driving force due to very large difference in contact angle by almost 15° in comparison to HA-4C-5Ce-5Ag) may be more than the combined effect of hydrophobicity and surface chemistry as in HA-4C-5Ce-5Ag.

### 3.5. Antibacterial Efficacy of Silver-Reinforced HA Coating

**Figure 9:**
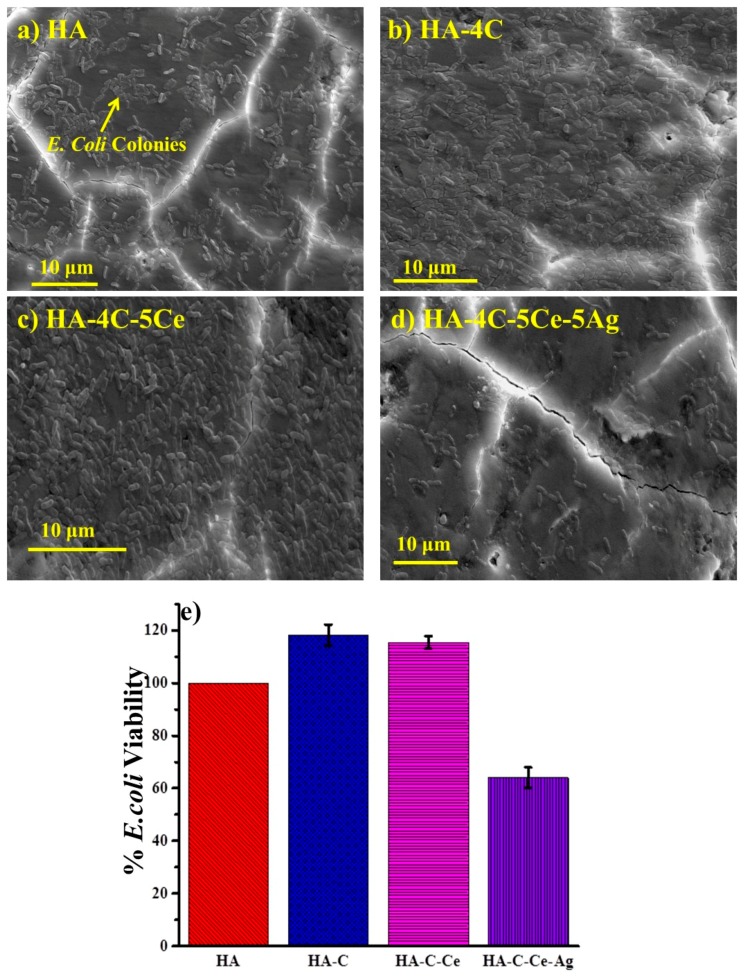
Scanning electron micrographs (**a**–**d**) and quantitative estimation (**e**) of *E. coli* on HA-based plasma sprayed coatings.

The adhesion of *E. coli* bacteria on HA-based coatings are shown in Figure 9a–d after 4 h incubation time. The scanning electron micrographs shows that when compared to HA (Figure 9a), the bacterial adhesion increases by CNT addition (Figure 9b), which has also been reported by Herkendell et al. [22]. A similar bacterial adhesion on HA-4C-Ce reveals the inert nature of CeO_2_ toward *E. coli* (Figure 9c). The least bacterial adhesion was observed on HA-4C-5Ce-Ag sample, Figure 9d, due to the silver reinforcement, attributed to its bactericidal role. The -SH group of many enzymes are targeted by Ag ions, thereupon restricting protein synthesis. Furthermore, the bacterial DNA is also denatured by Ag, thereby killing the bacteria [19].

The quantitative estimation of live bacteria, by MTT assay (Figure 9e), also correlated well with the bacterial density in the SEM images (Figure 9a–d). In comparison to pure HA coating, HA-4C represents 118.24 ± 3.97% higher bacterial adhesion. The dense *E. coli* colonies on the HA-4C sample confirms the role of CNT assisting in the *E. coli* adhesion and proliferation also reported by [22]. While considering the composite HA-4C-5Ce, an almost similar (115.4 ± 2.43%) bacterial density is obtained as in the case of HA-4C (118.24 ± 3.97%). The inert nature of CeO_2_ toward *E. coli* has also been reported earlier [23,58]. The character of silver is evident considering the least bacterial adhesion (64.04 ± 3.97%) on the HA-4C-5Ce-Ag composite coating. The -SH group of many enzymes are targeted by the permeating Ag ions through the bacterial membrane, thereupon restricting protein synthesis. Furthermore, the bacterial DNA is also denatured by Ag, subsequently causing bacterial death [19]. 

### 3.6. Cytocompatibilty Test

**Figure 10:**
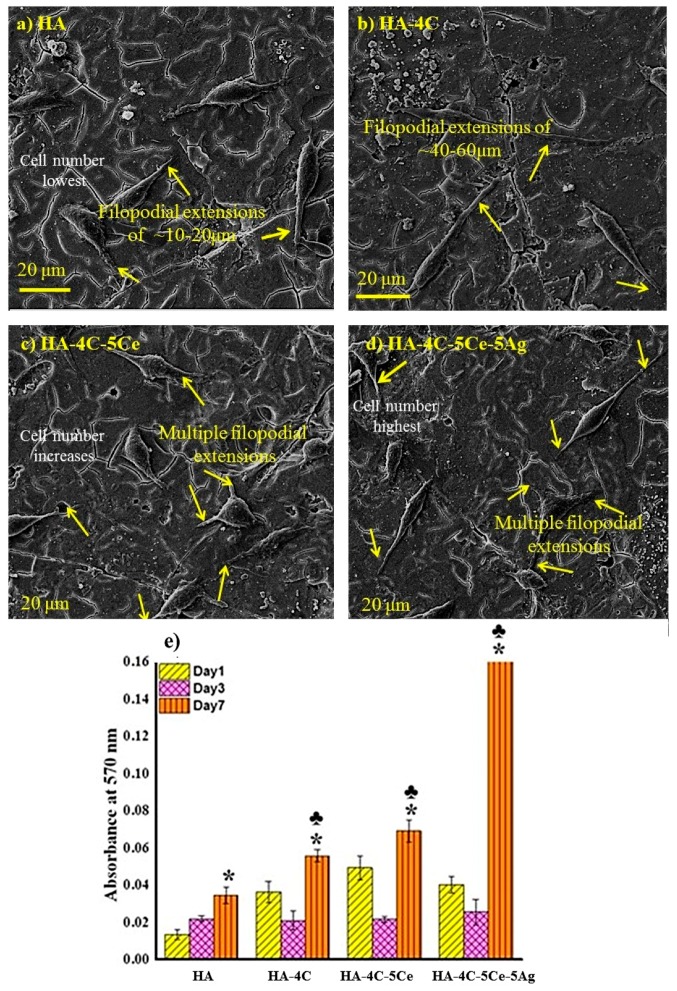
Scanning electron micrographs (**a**–**d**) and quantitative estimation (**e**) of L929 cells on HA-based plasma sprayed coatings. * Indicates *p* values of <0.007 for cell density average at day 7 of composites when compared to day 7 HA control. The average of the obtained cell number on day 7 composites in comparison to that of same composites on day 1 and ♣ depicts *p* value of <0.001.

It can be observed from the SEM images (Figure 10a–d) of the cell seeded scaffolds that the pure HA coating (Figure 10a) shows elongated structures called filopodia of 10–20 μm in length that appear to extend into filopodia-like-extensions ranging from 40–60 μm, with CNT reinforcement (Figure 10b), and further develop into more complex actin cytoplasmic connections (multiple filopodia developed from more than direction) with CeO_2_ (Figure 10c) and/or Ag (Figure 10d) reinforcement. This confirms the supporting nature of reinforcement materials (used in the coatings) towards the cell adhesion process. In other words, the HA-4C-5Ce-Ag loaded supports acted as a better microenvironment for L929 cells (stretched with filopodia and cytoplasmic extensions) than the pure HA coating, which is confirmed by the proliferation experiment.

Figure 10e presents the MTT assay results carried out with the L929 cells cultured on the pure HA and composite plasma sprayed coatings. Cell adhesion and proliferation after day 1 revealed an enhancement in cellular viability (majorly due to the fetal bovine serum protein adsorption from the media during the incubation period, overnight) on all the composite coatings in comparison to the pure HA coating. It can be noticed that the highest number of cells were obtained on HA-4C-5Ce construct (3.7 times than that of HA), which relates to the highest protein adsorption on the same (Table 3), which is attributed to the hydrophobic effect of CNT and CeO_2_. The cell count on HA-4C-Ce-Ag sample (3.0 times higher relative to HA) was higher than HA-4C (2.7 times higher relative to HA), again following the similar trend of the protein adsorption.

However, the day 2 readings show a downfall of the cell number on the samples containing CNT, CeO_2_, or Ag due to their hydrophobic nature, now governing the cell interaction process. This is because of the occurring protein adsorption up to 48 h, after which the desorbed protein so not play a role in the cell-interaction process, now leaving the cell-adhesion to be controlled by the surface properties of the samples [59]. The increment in cell numbers when compared to day 1 of the same sample was achieved maximum for pure HA (1.6 times higher than day 1 HA). There was an observed cell density decrease on the hydrophobic HA-4C-5Ce-Ag (1.6 times lower than day 1 HA-4C-5Ce-Ag) and HA-4C (1.7 times lesser than day 1 HA-4C), with the maximum decrement on the most hydrophobic HA-4C-5Ce (2.3 times lesser than day 1 HA-4C-5Ce) coating. Peculiarly, the cell numbers on HA, HA-4C and HA-4C-5Ce were found to almost in the same range, with the best density on HA-4C-5Ce-Ag, indicating it to support cell adhesion and proliferation in comparison to all the remaining coatings, as surface is well-covered (confluent) with cells.

The day 7 reading, nonetheless, increased the cell densities on all the substrates when compared to day 1. Besides, the cell numbers on the all the reinforced samples was higher than pure HA day 7. This is due to the physiological activities like extra cellular matrix mineralization of the cells, after 72 h (post-mitotic period), during which the actual surface properties of substrate may not influence cellular adhesion or proliferation [60].

Although cell proliferation was high on all composite supports, throughout the incubation period, the largest densities were collected on the HA-4C-5Ce-Ag loaded constructs (4.8 times higher, attributed to the synergistic effect of CNT, CeO_2_ and Ag), followed by HA-4C-Ce coated sample increased 2.0 times and HA-4C increased 1.6 times relative to HA day 7. The cell density is found to increase with CeO_2_ (resemblance with Ca^2+^ assisting in cell growth [61,62]) and Ag (the low/nil cytotoxicity of Ag when used in low concentration [22]) reinforcement, as already stated elsewhere [23]. The cell number on the HA-4C sample was found to be the least amongst the composite coatings, however this (CNT reinforced HA coated sample) gave rise to much better cell adhesion and proliferation (1.6 times) when compared to pure HA coating which can be attributed to the inclining properties of the CNTs toward cell growth as reported by [22,25,32]. This confirms that the HA-4C-5Ce-Ag to support cell growth and is an optimal substrate for its use in orthopedic applications.

**Figure 11:**
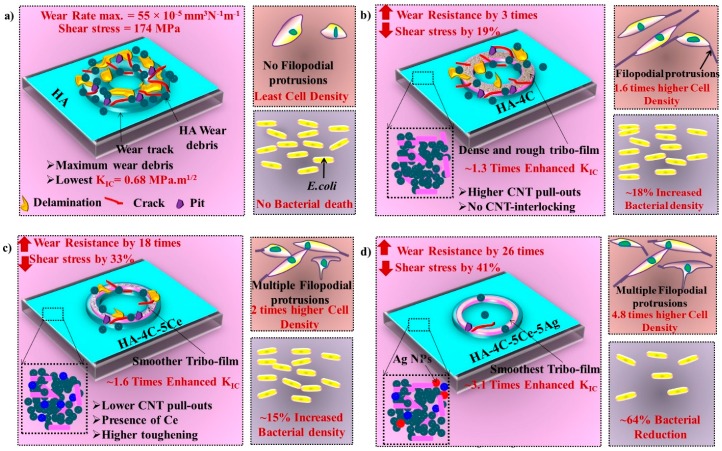
Schematic representing the plausible mechanisms involved for tribological resistance and fracture toughness, cell density, and bacterial adhesion on (**a**) HA-, (**b**) HA-4C-, (**c**) HA-4C-5Ce-, and (**d**) HA-4C-5Ce-5Ag-coated TiAl6V4 substrate.

The schematic in Figure 11 presents the mechanical, tribological, and biological properties of HA-based plasma-sprayed coatings on TiAl6V4 substrate. The pure HA coating (Figure 11a) shows the lowest mechanical property (toughness of 0.68 MPa·m^1/2^, Table 1) and therefore the least tribological resistance (wear rate of 176.1 × 10^−5^ mm^3^·N^−1^·m^−1^, Table 2), the least L929 cell density, and no antibacterial property. For the CNT incorporated HA-4C coating (Figure 11b), the increased toughness by 1.3 times (Table 1) and the CNT pull-outs from the matrix led to the formation of graphitic tribo-film (Figure 6b), tribological resistance by 3 times (Table 2) with 1.6 times higher cell growth (with filopodial protrusions) and 18% higher bacterial growth. The fracture toughness (attributed to the crack bridging by CNTs and toughening by CeO_2_ NPs) and tribological resistance by 1.6 times and 18 times higher, respectively, while cell density (with multiple filopodial protrusions) was enhanced by 2 times but with 15% higher bacterial growth (Figure 11c). Figure 11d (HA-4C-5Ce-5Ag) shows 3.1 times higher toughness, 26 times higher wear resistance, 4.8 times elevated cell density, and 46% bacterial reduction (in comparison to HA coating). The fracture toughness is enhanced by the synergistic effect of CNT pull-outs and crack bridging, and volumetric toughening provided by CeO_2_ and Ag NPs. There was increased cell density again due to synergy between the reinforcements, leading to development of multiple filopodial protrusions (of ~40 μm). The flat and elongated cells further confirm the contact-intimacy with the coating. The bactericidal Ag NPs lead to decrement in *E. coli* density due to denaturing DNA and the proteins. Thus, CNT, CeO_2_ and Ag incorporated HA coating leads to enhanced mechanical, cytocompatible, and antibacterial efficacy.

## 4. Conclusions

The HA-based coatings were plasma-sprayed on TiAl6V4, and the obtained coating thickness was 100–130 µm. HA-CNT-CeO_2_-Ag plasma-sprayed coating elicited a tribological resistance of 28 times more than that of pure HA coating attributed to the combined effect of deposited graphitic tribo-film and enhanced mechanical properties (2.3 times 1.6 times, and 3.1 times enhanced Vickers hardness, estimated modulus, and fracture toughness, respectively). The resulting shear stress for HA coating was estimated to be maximum (173.7 MPa), which reduced to 102.9 MPa with CNT-CeO_2_-Ag reinforcement. The Archard’s wear constant was computed to be decreased by an order of magnitude for HA-CNT-CeO_2_ and HA-CNT-CeO_2_-Ag, which portrays the wear resistance being governed by the hardness of the material. Furthermore, the highest stress (due to a maximum of 1.1 GPa Hertzian contact pressure and 0.16 COF amongst coatings) generated, for HA, consequently led to maximum worn volume (30 times more than HA-4C-4Ce-5Ag). The 19% lower stress generated for the CNT-reinforced coatings due to lubrication from graphitization and the higher mechanical property (*H*, *K_IC_*) led to a 3 times decrement (than HA) in the worn volume. The combined action of lowest Hertzian contact stresses (33% for HA-CNT-CeO_2_ and 41% for HA-CNT-CeO_2_-Ag, relative to HA) and the highest hardness and toughness resulted in 20 and 30 times reduction of wear volume for CeO_2_- and CeO_2_-Ag-incorporated HA-CNT coatings, respectively. The composite coating was found to be bactericidal for *E. coli* (46% reduced bacterial adhesion), elicited by the Ag reinforcement. Maximum fibroblast adhesion was attained on HA-4C-5Ce-5Ag construct on day 7 due to the synergistic effect of reinforcing materials, concluding the non-toxicity of the used CNTs, CeO_2_, and Ag (in lower concentrations) toward the L929 cell line. Hence, it is concluded that the HA-CNT-CeO_2_-Ag plasma-sprayed coating successfully enhanced the wear resistance due to enhanced hardness and toughness (accredited to synergistic role of reinforcements) and was also found to be cytocompatible and bactericidal, thus making it a potential substrate for hard-tissue replacements.

## Figures and Tables

**Table 1:** Abbreviations used and the mechanical properties (*H*, *E*, and *K_IC_*) of the coatings.

Plasma Sprayed Coatings	Abbreviations	*H* (GPa)	*E*/*H*	*K_IC_* (MPa·m^1/2^)
Hydroxyapatite	HA	1.88 ± 0.88	44	0.68
Hydroxyapatite-CNT	HA-4C	3.95 ± 0.96	41.8	0.89
Hydroxyapatite-CNT-CeO_2_	HA-4C-5Ce	4.13 ± 0.54	41.4	1.12
Hydroxyapatite-CNT-CeO_2_-Ag	HA-4C-5Ce-5Ag	5.85 ± 0.85	29.3	2.12

**Table 2:** Characterization from ball on disc test of HA-based plasma-sprayed samples.

Sample	COF	W_R_ (×10^−6^ mm^3^·N^−1^·m^−1^)	Roughness after Wear (µm)	E* (GPa)	HCD (µm)	*τ* (MPa)	Wear Resistance
HA	0.16 ± 0.01	176.1 ± 7.0	12	77.1	66.3	173.7	Base
HA-4C	0.11 ± 0.01	54.7 ± 5.2	10	98.5	61.1	140.6	3.2 times
HA-4C-5Ce	0.09 ± 0.01	9.7 ± 0.8	6	100.3	61.7	115	18.1 times
HA-4C-5Ce-5Ag	0.08 ± 0.01	6.8 ± 0.2	5	99.5	61.9	102.9	25.9 times

**Table 3:** Water contact angles (WCA), the corresponding BSA adsorption, and cell density improvement on the HA-based coatings (* indicates *p* values of mean adsorbed proteins on composites when compared to HA control: <0.0001).

Sample	Contact Angle (Degree)	Protein Adsorption (μg/mm2)	Day 1 Cell Density Improvement
HA	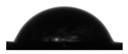 74.0° ± 1.8	2.1 ± 0.1	1
HA-4C	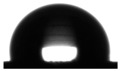 104.0° ± 1.1	3.4 ± 0.1 *	2.7 times
HA-4C-5Ce	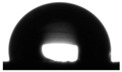 112.0° ± 1.3	5.0 ± 0.1 *	3.7 times
HA-4C-5Ce-5Ag	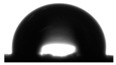 97.0° ± 2.0	±0.1 *	3.0 times
